# Antibacterial and Photocatalytic Performance of EPS-Stabilized Silver Nanoparticles Immobilized on Activated Natural Zeolite

**DOI:** 10.3390/polym18151868

**Published:** 2026-07-30

**Authors:** Sergio Benavides-Valenzuela, Enzo Pagueguy, Rodrigo Segura, Aparna Banerjee, Shrabana Sarkar

**Affiliations:** 1 Facultad de Ciencias de la Rehabilitación y Calidad de Vida, Universidad San Sebastián, Concepción 4080871, Chile; sergio.benavides@uss.cl (S.B.-V.); epagueguyr@correo.uss.cl (E.P.); 2 Instituto de Química, Facultad de Ciencias, Universidad de Valparaíso, Valparaíso 2340000, Chile; rodrigo.segura@uv.cl; 3 Functional Polysaccharides Research Group, Instituto de Ciencias Aplicadas, Facultad de Ingeniería, Universidad Autónoma de Chile, Sede Talca, Talca 3460000, Chile

**Keywords:** biogenic silver nanoparticle, natural zeolite, antimicrobial activity, photocatalytic degradation

## Abstract

Biopolymers have emerged as effective stabilizing and capping agents in the green synthesis of metal nanoparticles due to their biocompatible, hydrophilic, and eco-friendly nature. Among these, bacterial polysaccharides, particularly exopolysaccharides (EPSs), offer distinct advantages over plant, algal, and fungal derived polymers, owing to their unique chemical structure rich in negatively charged functional groups that facilitate interactions with metal ions and enhance nanoparticles’ stability. Despite their properties, the practical application of AgNPs is often limited by aggregation, colloidal instability, and uncontrolled ion release. To address these challenges, in the present study, EPS-stabilized silver nanoparticles (AgNPs) were synthesized using a green approach. The synthesized biogenic AgNPs were immobilized with natural zeolite, a porous inorganic material, to improve stability and regulate functionality. The resulting catalytic material was partially characterized using structurally using spectrophotometry (UV-Vis), scanning electron microscope - energy dispersive X-ray analysis (SEM-EDAX), dynamic light scattering (DLS), and Fourier-transform infrared spectroscopy (FTIR). Furthermore, the synthesized green catalyst was evaluated for its photocatalytic degradation against synthetic dyes (methylene blue). In addition, its antibacterial potential was also assessed through determination of inhibitory concentration (IC_90_) and minimum bactericidal concentration (MBC_99_), highlighting its potential as a multifunctional biomaterial for environmental and biomedical applications.

## 1. Introduction

Nanotechnology has provided new opportunities for the design of active materials with high surface area, adaptable surface chemistry, and enhanced interaction capacity for environmental and biomedical applications. Among these, metallic nanoparticles have attracted considerable attention due to their nanoscale dimensions, high surface reactivity, and unique physicochemical properties. In particular, among different metals, silver (Ag) has been widely used since antiquity for water and food preservation, as a therapeutic agent, as well as for therapeutic purposes because of its well-established antimicrobial activity [1,2,3]. At the nanoscale, silver nanoparticles (AgNPs) exhibit enhanced biological and catalytic performance, which is largely associated with their high reactive surface area and their ability to release Ag^+^ ions under aqueous conditions [4,5]. These characteristics make AgNPs highly promising for antimicrobial application and organic contaminant removal. Despite these advantages, conventional physical and chemical methods often require high energy input, toxic solvents, strong reducing agents, or synthetic stabilizers, which may limit their environmental compatibility and sustainability. Consequently, green synthesis approaches have emerged as attractive alternatives for nanoparticle production under relatively mild and environmentally friendly conditions [6]. Biological matrices are relevant as they can simultaneously mediate metal ion reduction, nucleation, growth control, and colloidal stabilization during the formation of nanoparticles [6]. In this context, biomolecules such as polysaccharides, proteins, phenolic compounds, amino acids, organic acids, and enzymes can mediate nanoparticle formation and generate surface capping layers that influence size distribution, surface charge, aggregation behavior, and biological interactions [7].

Among these biomolecules, polysaccharides have gained special attention due to their structural diversity and stabilization capacity. The chemical and morphological structure of the polymeric chain also helps to stabilize the synthesis as well as dispersion, acting as stabilizing/capping agents in the green synthesis of metal nanoparticles [8]. Scheffel et al. [9] first investigated the synthesis of biopolymeric nanoparticles, using the natural polymer albumin, while more recent studies focus on biodegradable polymers as a sustainable choice due to their non-toxic, hydrophilic, and eco-friendly nature [10,11]. Compared to plant-, algal-, and fungal-origin polysaccharides, bacterial polysaccharides are advantageous due to their unique chemical composition and structural functionality. Their negative charge through the presence of carboxylic, phosphoric, or sulfate functional groups, and interactive positively charged organic components [12,13] make them capable of binding metallic ions and interacting with nanoparticles’ surfaces [14]. Therefore, bacterial exopolysaccharide (EPS) can act not only as capping agents but also as hydrated bioorganic interfaces that provide steric and electrostatic stabilization. This feature is particularly relevant for environmental applications, where nanoparticle aggregation and instability may affect catalytic performance and environmental safety. These unique physicochemical characteristics of bacterial EPSs make them suitable for the development of nanocatalytic systems based on metallic nanoparticles. In addition to biopolymer stabilization, the incorporation of minerals has emerged as an effective strategy to overcome some of the limitations associated with free nanoparticles, including aggregation, uncontrolled dispersion, and recovery difficulties. Among mineral materials, natural zeolite has received attention due to its aluminosilicate framework, high surface area, ion-exchange capacity, chemical stability, porous structure, and affinity for different contaminants [15]. These characteristics make zeolite an efficient support material for adsorption and immobilization processes. When combined with EPS-stabilized AgNPs, zeolite can act as an inorganic carrier that restricts nanoparticle mobility, facilitates recovery or handling, and provides additional interaction sites for contaminant removal. In this hybrid configuration, the EPS layer may contribute to nanoparticle stabilization and metal–surface interaction, while the zeolite matrix may provide structural support, adsorption capacity, and reduced nanoparticle dispersion [16].

Based on these considerations, the present study aimed to obtain and characterize free and zeolite-supported EPS-stabilized silver nanoparticles and to evaluate their dual function as antimicrobial materials and synthetic dye removal in aqueous media. By integrating physicochemical characterization with biological and environmental application assays, this work seeks to clarify how EPS stabilization and zeolite immobilization influence the behavior and functionality of AgNP-based hybrid materials, supporting their potential use as a sustainable multifunctional platform for water treatment.

## 2. Materials and Methods

### 2.1. Bacterial Exopolysaccharides (EPSs)

Bacterial exopolysaccharide (EPS) was obtained from *Bacillus licheniformis* Tol1 [17]. The strain was cultivated in LB medium under controlled conditions at 55 °C for 3 days with agitation at 130 rpm. For extraction of EPSs, the culture broth was centrifuged at 10,000 rpm at 4 °C to remove bacterial cells. The cell-free supernatant was recovered and subjected to EPS precipitation by adding cold acetone (99.5%, Winkler Ltda., Santiago, Chile) and stored at 4 °C for 24 h. The precipitated crude EPS was recovered by centrifugation and lyophilized until using them as a stabilizing matrix for AgNP synthesis.

### 2.2. Green Synthesis of AgNPs and Their Partial Characterization

Biogenic synthesis of polysaccharide-stabilized AgNPs was performed using the co-precipitation method as described before by Jayabalan et al. [18] with required modifications. AgNO_3_ was used as a precursor, and bacterial EPS was used as a stabilizing/capping agent. For this, a 1 mM AgNO_3_ aqueous solution was added to the EPS solution (1 mg mL^−1^) in 1:1 (*v*/*v*) under sterile conditions. Each reaction mixture was incubated at 50 °C in the dark with continuous shaking at 130 rpm for 12 h. The synthesis was monitored by observing the change in color from pale yellow to greenish black, indicating the formation of the nanoparticle. The resulting colloidal suspension was centrifuged (8000 rpm, 15 min, 4 °C), double washed using deionized water, and lyophilized for further use.

The partial characterization of the green-synthesized biogenic nanoparticle was performed according to the method described before by Prakash et al. [19]. The UV–visible spectrum of the AgNP colloidal suspension was scanned between 200 and 800 nm using a FlexA-200 full-wavelength microplate reader (Allsheng Instruments Co., Ltd., Hangzhou, China), where an aqueous solution of AgNO_3_ was used as a control. The morphology, surface distribution, and microstructural features of free EPS-stabilized AgNPs were evaluated by field-emission scanning electron microscopy (FESEM) and scanning transmission electron microscopy in SEM mode (STEM-in-SEM) using a Quattro S ESEM system (Thermo Fisher Scientific, Waltham, MA, USA). For conventional SEM observation, dry samples were mounted onto aluminum stubs using conductive carbon adhesive tape and sputter-coated with a thin gold layer using a Leica ACE600 sputter coater (Wetzlar, Germany) when required to minimize charging effects. SEM micrographs were acquired using an Everhart–Thornley secondary electron detector (ETD) (Cambridge, UK) at an accelerating voltage of 30 kV. Images were obtained at different magnifications to observe particle morphology, aggregation state, and EPS-associated structures. To improve the nanoscale visualization of the silver nanoparticles and enable individual particle size assessment, complementary transmission-mode imaging was performed using a STEM3+ detector coupled to the FESEM system. For this analysis, the samples were dispersed 1:100 in deionized water and deposited onto holey carbon-coated copper grids. Bright-field (BF) and high-angle annular dark-field (HAADF) images were acquired at 30 kV. HAADF imaging was particularly useful for identifying electron-dense silver-rich nanostructures due to the enhanced atomic-number contrast of metallic silver. This approach allowed direct visualization of individual AgNPs and supported the assessment of their morphology and distribution. Particle diameters were estimated from clearly distinguishable individual nanoparticles observed in the STEM-in-SEM micrographs using image analysis software (https://www.thermofisher.com/cl/es/home/electron-microscopy/products/software-em-3d-vis/velox-software.html; accessed on 22 May 2026). Elemental analysis was performed by energy-dispersive X-ray spectroscopy (EDAX) using an UltraDry EDAX detector, 30 mm^2^, 129 eV Mn resolution, Thermo Fisher Scientific (Waltham, MA, USA). Representative regions from each sample were selected to confirm the presence and spatial distribution of silver in green-synthesized AgNP systems. The hydrodynamic size distribution and colloidal stability of the synthesized AgNPs were evaluated by dynamic light scattering (DLS) and electrophoretic light scattering (ELS), respectively, using a ZetaSizer Pro instrument (Malvern Panalytical, Malvern, UK) at 25 °C. For DLS analysis, a stock AgNP suspension (0.1 mg mL^−1^) was prepared in double-distilled water and further diluted prior to measurement in UV-grade cuvettes. For zeta potential analysis, samples were dispersed in ultrapure water and analyzed in disposable capillary cells. Zeta potential values were calculated using the Smoluchowski approximation.

### 2.3. Immobilization of Biogenic AgNPs in a Natural Zeolite Matrix

The AgNP/zeolite nanocomposite was prepared through a combined strategy involving thermal pretreatment, acid activation, wet impregnation, and subsequent photoinduced fixation [20]. Natural zeolite was first subjected to direct heating for 10 min to reduce the organic load, since organic residues may block zeolitic pores and impair adsorption performance. The material was then calcined in a muffle furnace at 550 °C for 1 h to remove volatile compounds and thermally activate the mineral matrix [21]. Subsequently, the calcined zeolite was treated with HCl (1 M) under continuous stirring for 24 h to promote acid activation and ion exchange. This step was intended to remove exchangeable cations and residual impurities, increase the availability of surface silanol and acidic sites, and enhance the interaction capacity of the zeolitic surface [20]. After acid activation, the solid material was repeatedly washed with deionized water until it reached a neutral pH, then it was recovered by centrifugation and dried at 60 °C until a constant weight was achieved. Zeolite-supported EPS-stabilized AgNPs were then prepared by wet impregnation. Then, 2 g of dried, activated zeolite was dispersed in 10 mL of an aqueous EPS-stabilized AgNP suspension containing 10 mg mL^−1^ AgNPs, corresponding to an initial zeolite-to-AgNP suspension ratio of 200 mg mL^−1^ and a total AgNP input of 100 mg. The mixture was maintained under continuous stirring for a fixed time at a controlled temperature to promote contact between the nanoparticles and the zeolitic surface. Intermittent mild sonication was applied to improve nanoparticle dispersion and favor their interaction with mineral support.

To improve nanoparticle retention, the dried Zeo-AgNP material was subjected to UV-C-assisted fixation [22]. Samples were spread as a thin layer and irradiated at 254 nm for 60 min at a distance of 10–15 cm from the lamp, with manual turning after 30 min to ensure homogeneous exposure. After UV-C treatment, the material was additionally dried at 60 °C for 4 h to promote post-fixation stabilization of the immobilized nanoparticles. Activated zeolite without AgNPs was processed under the same conditions and used as matrix control. The final materials were stored in sterile, light-protected containers until characterization and functional assays. The interfacial reaction is as follows (Equation (1)):

(1) $$M-OH_{surf}+HO-Si\equiv \to \left(h\gamma \right)\to M-O-Si\equiv +\;H2O\;$$

where *M* represents Ag.

### 2.4. Partial Characterization of Zeolite Immobilized EPS-Stabilized AgNPs (Zeo-AgNPs)

#### 2.4.1. Scanning Electron Microscopy and Elemental Analysis

The morphology and surface distribution were evaluated by scanning electron microscopy (SEM). Dry samples were mounted onto aluminum stubs using carbon adhesive tape and coated with a thin conductive layer of gold. Images were acquired at different magnifications to observe particle morphology, aggregation state, EPS-associated structures, and the deposition of AgNPs on the zeolite surface. Elemental analysis was performed by energy-dispersive X-ray spectroscopy (EDAX) coupled to SEM. This analysis was used to confirm the presence and spatial distribution of silver in both free and zeolite-immobilized AgNP systems. For zeolite-supported materials, SEM–EDAX analysis also allowed evaluation of the association of silver-containing domains with the zeolitic matrix and comparison with unloaded zeolite controls.

#### 2.4.2. Fourier-Transform Infrared Spectroscopy (FTIR) Analysis

Fourier-transform infrared spectroscopy (FTIR) (Perkin Elmer, Model Spectrum Two, Waltham, MA, USA) was used to identify functional groups involved in the reaction and stabilization. Comparative spectra of activated zeolite and nanoparticle-loaded samples (Zeo-AgNPs) were used to evaluate structural modifications after nanoparticle immobilization. It was analyzed in the spectral range of 4000–400 cm^−1^. Spectra were obtained using attenuated total reflectance (ATR) mode. The spectra were compared to identify shifts, appearance, or attenuation of characteristic bands associated with hydroxyl, carboxyl, carbonyl, amine, glycosidic, and aluminosilicate framework vibrations. These spectral changes were interpreted as indirect evidence of EPS-mediated AgNP stabilization and interaction of AgNPs-EPS with the zeolitic support.

#### 2.4.3. Hydrodynamic Diameter, PDI and Zeta Potential Analysis

The hydrodynamic diameter, polydispersity index (PDI), and zeta potential of Zeo-AgNPs were determined by dynamic light scattering (DLS) and electrophoretic light scattering, respectively. Prior to analysis, samples were dispersed in sterile deionized water and homogenized by vortexing and mild sonication to reduce aggregation. Measurements were performed at room temperature using disposable cuvettes for DLS and folded capillary cells for zeta potential analysis. For each formulation, at least three independent measurements were recorded. Hydrodynamic diameter and PDI were used to evaluate colloidal size distribution and dispersion homogeneity, whereas zeta potential was used as an indicator of surface charge and colloidal stability. In the case of zeolite-supported systems, the results were interpreted considering the larger size of the mineral carrier and the possible contribution of zeolite-associated aggregates.

### 2.5. Evaluation of Antibacterial Activity and Determination of IC_90_ and MBC_99_

#### 2.5.1. Preliminary Qualitative Evaluation by Agar Diffusion Assay

The antibacterial activity of free EPS-stabilized AgNPs and Zeo-AgNPs was initially evaluated using an agar diffusion assay as a preliminary qualitative screening. Briefly, suspensions of *Escherichia coli*, *Listeria monocytogenes*, *Salmonella typhi*, and *Staphylococcus aureus*, previously grown to the logarithmic phase, adjusted to approximately 10^6^ CFU mL^−1^ and surface-inoculated onto tryptic soy agar plates (HiMedia™, Mumbai, India). A total of 0.3 mg of each sample was used for the analysis. Commercial antibiotic disks were used as positive controls, whereas activated zeolite without AgNPs and EPS solution were used as negative controls. Inoculated plates were incubated at 37 °C for 24 h, and antibacterial activity was qualitatively assessed based on the presence or absence of inhibition zones (inhibition halo diameters in mm).

#### 2.5.2. Determination of IC_90_ and MBC_99_

The IC_90_ and MBC_99_ values of free EPS-stabilized AgNPs and Zeo-AgNPs were determined by broth microdilution methods using sterile 96-well microplates. The same four bacterial strains evaluated in the preliminary agar diffusion assay (*Escherichia coli, Listeria monocytogenes, Salmonella typhi, and Staphylococcus aureus*) were used for quantitative assays. Lyophilized samples were dissolved in sterile distilled water and subjected to serial two-fold dilutions. Each well contained tryptic soy broth, bacterial inoculum (10^6^ CFU mL^−1)^, and the corresponding sample solution (AgNPs, Zeo-AgNPs) at the selected concentration. Regarding the control bacterial growth, only media, zeolite-only control, EPS control, and optical interference blanks were included for each formulation. Microplates were incubated at 37 °C for 24 h, and the bacterial growth was determined by measuring absorbance at 590 nm. The IC_90_ was defined as the concentration required to reduce bacterial growth by 90% in comparison to the untreated growth control. To confirm bactericidal activity, aliquots from treated wells were plated onto tryptic soy agar and incubated at 37 °C for 24 h. The MBC_99_ was defined as the lowest concentration producing approximately 99% reduction in viable bacterial counts in comparison to the untreated control. The MBC_99_/IC_90_ ratio was calculated to evaluate whether the antibacterial activity was predominantly bactericidal or bacteriostatic.

### 2.6. Synthetic Dye Removal Assay Using Zeo-AgNPs

Photocatalytic decolorization was done as stated before by Vanaamudan et al. [23], Das and Dhar [24], and Sarkar et al. [25]. The reaction mixture was prepared by mixing 5 mL of Zeo-AgNP stock solution (1 mg ml^−1^ and 2 mg ml^−1^), 10 mL of methylene blue (MB) (HiMedia^TM^, India) solution (100 and 200 mg L^−1^), and 5 mL of 10% (*v*/*v*) H_2_O_2_ (HiMedia^TM^, India). An aqueous solution of MB with H_2_O_2_ without the addition of Zeo-AgNPs was used as a control. All the experimental sets were incubated under full sunlight for 4 h, and the final absorbance was measured to calculate the % of decolorization using Equation (2), where *C*_0_ is the initial absorbance and *C_t_* is the final absorbance.

(2) $$\mathrm{Decolorization}\;\left(\%\right)=\frac{C_{0}-C_{t}}{C_{0}}\times 100$$

All experiments were performed at least in triplicate unless otherwise stated. Results were expressed as mean ± standard deviation (SD). Statistical differences among treatments were analyzed by one-way analysis of variance followed by Tukey’s post hoc test, with significance established at *p* < 0.05. Bio-transformed MB product after treating with AgNPs was studied using spectroscopy.

## 3. Results and Discussions

### 3.1. Characterization of Green Synthesis of Silver Nanoparticles (AgNPs)

**Figure 1:**
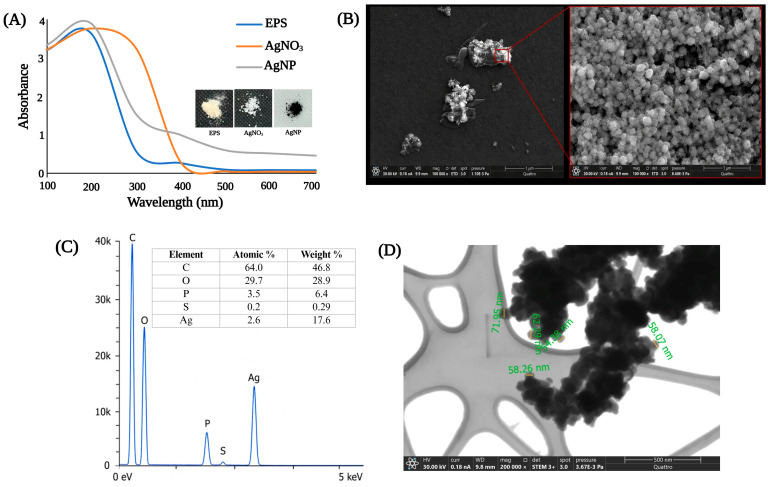
(**A**) UV–Vis spectroscopy analysis of EPS, AgNO_3_, and EPS-stabilized nanoparticles, along with the synthesized nanoparticle sample in powder form shown in the inset. (**B**) SEM micrograph showing the morphology of the EPS-stabilized AgNPs. (**C**) The EDAX spectrum confirming the presence of silver in the EPS-stabilized AgNP sample, together with carbon and oxygen associated signals attributable to the EPS-derived organic matrix. (**D**) The TEM micrograph showing the size of the EPS-stabilized AgNPs, ~60.99 nm for a single particle.

Primarily, the EPS-stabilized nanoparticles synthesized were confirmed by a variation in the color of the solution, from transparent to yellowish to brownish black at the end of the reaction. A UV-Vis spectrophotometer was used for the determination of optical properties of polysaccharide-stabilized AgNPs (Figure 1A). The formation was followed by measuring the surface plasmon resonance (SPR) over the range of 200–500 nm. The studies show that AgNPs contribute to the absorption around 350–500 nm, with the most representative region around 400–420 nm, primarily confirming the formation of nanoparticles. Similar kinds of studies have already been conducted by Asif et al. [26] about the green-synthesized AgNPs, where the absorption was noticed in the range of 390–470 nm (~419 nm). This observation also confirms the reduction of Ag^2+^ ions to Ag^0^ at the time of nanoparticle synthesis [27].

The structural organization of the synthesized biogenic AgNPs was evaluated by SEM coupled with elemental analysis. SEM micrographs revealed the formation of a heterogeneous nanoparticulate system composed mainly of nanoscale particles with variable size and morphology. Individual AgNPs were observed in the approximate range of 10–30 nm. The particles exhibited irregular spatial distribution, with quasi-spherical and non-uniform morphologies. At lower magnification (Figure 1B), the material appeared as discrete clustered domains distributed over the substrate surface rather than as a continuous compact layer, indicating localized aggregation. Overall, these observations indicate that the biogenic synthesis produced AgNPs with heterogeneous nanoscale organization and a tendency toward probable partial aggregation/agglomeration on the substrate surface. This heterogeneous morphology may be associated with the function of biomolecules involved in the nanoparticle synthesis along with their size and shape [28,29]. On the other hand, analysis in higher magnification (Figure 1B) also showed that the aggregates were composed predominantly of quasi-spherical and irregular nanoscale domains with variable particle dimensions, as found before by Chairil and Malmstadt [30]. The observed morphology is consistent with the formation of nanoparticle-associated clusters embedded within or coated by the EPS matrix. In several regions, individual rounded structures could still be distinguished despite the presence of aggregation, suggesting that the EPS layer may contribute to limiting complete particle union during drying and sample preparation. In addition, the EDAX spectrum (Figure 1C) prominently features the peak of Ag at ~2.5 keV, confirming its significant presence at 17.6 wt%. Apart from this, the EDAX spectrum also showed signals for oxygen (28.9 wt%) at ~0.5 keV and for carbon (46.8 wt%) at ~0.25 keV, and it is crucial for synthesis as well as the stabilization of nanoparticles, as also found before by Vinayagam et al. [31]. Overall, the microscopic observations coupled with EDAX spectrum analysis support the successful formation of EPS-stabilized AgNPs with nanoscale organization and heterogeneous aggregation behavior. The presence of densely packed particulate domains suggests that the EPS matrix acts as a stabilizing organic environment capable of maintaining particulate organization and restricting uncontrolled collapse into fully fused structures. Moreover, transmission electron microscopy (TEM) analysis validated nanoparticle formation and provided a more accurate assessment of particle size. It revealed predominantly quasi-spherical nanoparticles having a range between ~54-71 nm, with an average diameter of 60.99 ± 6.74 nm (Figure 1D).

**Figure 2:**
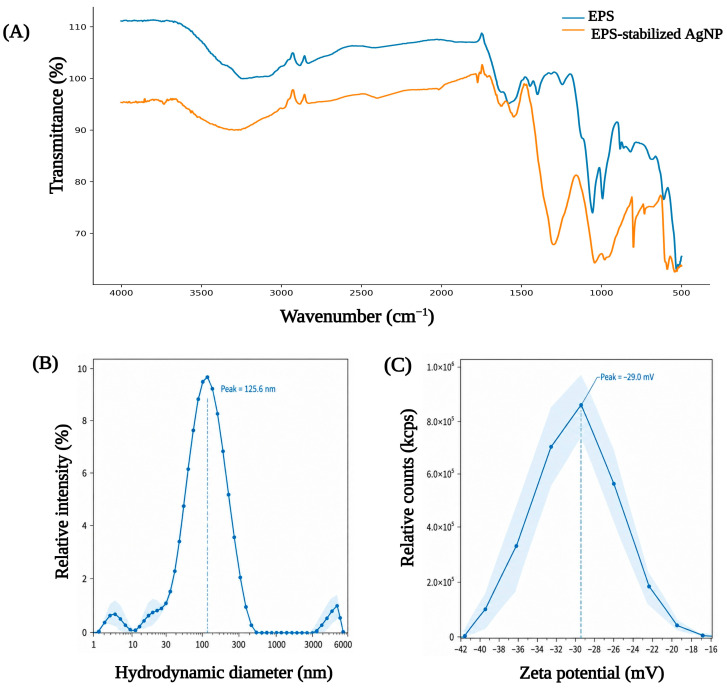
(**A**) Comparative FTIR spectra of EPSs and EPS-stabilized AgNPs were baseline-corrected, converted to apparent absorbance, min–max normalized, and vertically offset for clarity. (**B**) DLS profile showing a main population at ~125.6 nm. (**C**) Zeta potential distribution showing a dominant negatively charged population centered at ~−29 mV.

FTIR spectrum (Figure 2A) analysis revealed the functional groups of EPSs responsible for the reduction of silver ions for stabilizing the nanoparticle. A similar kind of study has been done before by Banerjee et al. [32]. The FTIR profile of the EPSs revealed the peaks in regions like 2900 cm^−1^ (-C-H- stretching), 1000–1200 cm^−1^ (-C-O- of glycosidic bonds) [33], 944–1030 cm^−1^ (stretching responsible for glucose), and 900–1100 cm^−1^ (stretching vibration responsible for sugar for polysaccharide nature) [34]. On the other hand, the FTIR spectrum of the EPS-stabilized AgNPs revealed peaks in regions like 3456 cm^−1^ (O-H stretching vibration), 1388 cm^−1^ (CH_3_ stretching vibration), 800 cm^−1^ (NH_2_ stretching vibration), and 617 cm^−1^ (-N-H stretching vibration), suggesting the participation of proteinaceous or amino-containing biomolecules in nanoparticle capping. Several characteristic EPS bands exhibited shifts in different positions responsible for changes in the intensity after AgNP formation, indicating the involvement of these functional groups in the synthesis and stabilization of nanoparticles. The broad O–H stretching band became more pronounced and shifted, suggesting interactions between hydroxyl groups and the AgNP surface. Likewise, changes observed in the glycosidic (C–O) and amino-containing regions indicate probable participation of oxygen- and nitrogen-containing functional groups in silver ion reduction and subsequent nanoparticle capping. Based on the shifting and changes, it is proposed that hydroxyl, glycosidic, and amino groups of the EPSs probably acted synergistically as reducing and capping agents for the synthesis of nanoparticles. Moreover, during nanoparticle formation, these functional groups facilitate the reduction of silver ions and subsequently adsorb onto the nanoparticle surface, forming a protective organic layer that prevents excessive aggregation and enhances colloidal stability. Similar observations have been reported in previous studies where hydroxyl, amino, and glycosidic groups of microbial EPSs acted as reducing and capping agents during biogenic nanoparticle synthesis [13].

The crystalline AgNPs showed maximum population distribution with a mean Z-average size distribution of 125.6 nm (Figure 2B), with a minimum particle size of ~30 nm and a smaller population within the 1–10 nm range, confirming the formation of nanoparticles having a zeta potential of −29.5 mV (Figure 2C). There is a similar kind of study where the Z-average value showed >100 nm (122.7 nm) for copper nanoparticles synthesized from leaf extract of *Ocimum sanctum* [35]. According to another example, biogenic silver nano-rod-shaped particles were synthesized from wastewater-origin bacterial strain *Pseudochrobactrum* sp. C5 was in the 100–120 nm size range [36]. As reported before for polysaccharide-stabilized gold nanoparticles, time-dependent size distribution analysis showed a shift in the larger particle population toward ~135 nm, likely due to aggregation with time [37]. Based on the exported intensity-weighted DLS size-distribution data, the main nanoparticle population showed an estimated PDI of approximately 0.28, suggesting a moderately polydisperse colloidal system.

### 3.2. Immobilization of Biogenic AgNPs in the Natural Zeolite Matrix

The AgNP/zeolite nanocomposite was successfully synthesized by immobilizing EPS-stabilized AgNPs onto activated natural zeolite through UV-assisted fixation. The synthesized nanocomposite, Zeo-AgNP, was further used for characterization and functionality. Theoretical calculations indicated an AgNP loading of approximately 50 mg g^−1^ zeolite (5 wt%); however, the actual silver content and Ag^+^ release profile require further confirmation using ICP-based analytical techniques.

### 3.3. Partial Characterization of Zeolite Immobilized EPS-Stabilized Silver Nanoparticles (Zeo-AgNPs)

#### 3.3.1. Scanning Electron Microscopy (SEM) and Elemental Analysis

The characterization was performed to examine the preservation of zeolitic material after nanoparticle immobilization, assess the spatial distribution, and obtain complementary evidence supporting the successful immobilization of EPS-stabilized AgNPs onto the inorganic support. The SEM coupled with EDAX provided evidence supporting the successful immobilization of EPS-stabilized AgNPs onto the zeolitic matrix while preserving the structural integrity of the support material. SEM observations revealed that the Zeo-AgNP maintained a crystalline morphology characterized by elongated, prismatic, and faceted structures typical of aluminosilicate zeolitic materials (Figure 2A). The preservation of well-defined crystalline domains after nanoparticle immobilization suggests no major structural collapse or surface degradation of the support. This behavior is particularly relevant because the maintenance of the zeolitic architecture is directly associated with the increase in surface area, adsorption sites, and ion-exchange functionality, which are important for environmental remediation applications [38]. Although individual metallic nanoparticles cannot be proven resolved at this magnification due to partial aggregation and EPS coverage, the observed surface roughening and particulate decoration strongly suggest successful nanoparticle deposition onto the mineral carrier.

The EDAX spectrum complemented the morphological observations by confirming the elemental composition of the material. In the present study, the EDAX was investigated in the binding energy region of 0–5 keV (Figure 2B) for the Zeo-AgNP nanocomposite. The spectrum reveals a clear peak between 3 and 4 keV, assigned to Ag (19.4 wt%) evidence supporting the incorporation of silver nanoparticle structures (AgNPs) within the supported system. Moreover, the peaks in the region between 0 and 2 keV related to O (3.9 wt%), Al (0.8 wt%), and Si (75.9 wt%) indicate the presence of zeolite. The dominant silicon-associated signal, together with oxygen and aluminum contributions, is consistent with the expected aluminosilicate composition of the zeolite framework, as also found before by Badvi and Javanbakht [39]. However, the relatively lower intensity of the Ag signal compared with Si is expected for supported nanoparticle systems in which the metallic phase represents a minor surface-associated fraction relative to the bulk mineral matrix.

#### 3.3.2. Fourier-Transform Infrared Spectroscopy (FTIR) Analysis

**Figure 3:**
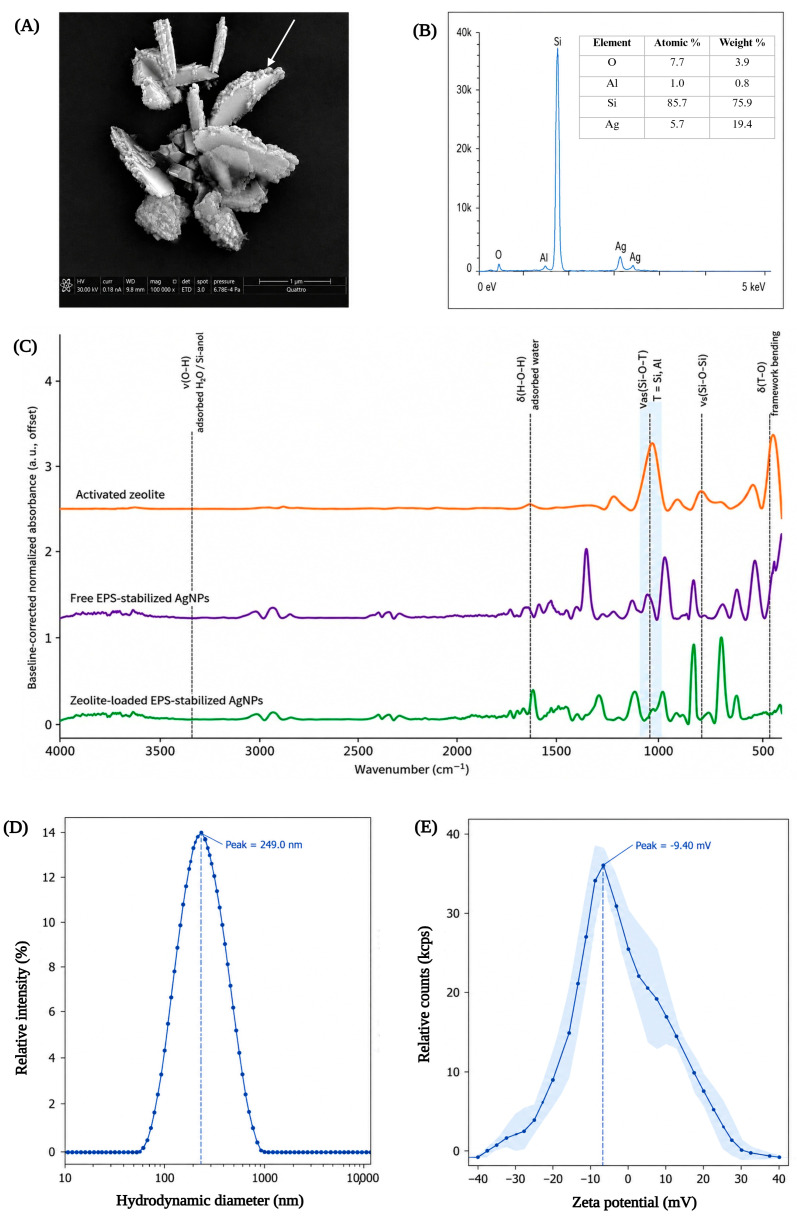
(**A**) SEM micrograph showing the crystalline morphology of the zeolitic support and the presence of heterogeneous surface-associated particulate domains compatible with immobilized EPS-stabilized AgNPs (marked with a white arrow). (**B**) EDAX spectrum confirming the aluminosilicate composition of the zeolite matrix and the presence of silver-associated signals in the hybrid material. (**C**) Comparative FTIR spectra of activated zeolite, EPS-stabilized AgNPs, and zeolite-supported EPS-stabilized AgNPs. Spectra were baseline-corrected, converted to apparent absorbance, min–max normalized, and vertically offset for clarity. The shaded region indicates the main zeolitic framework domain, whereas the spectral profile of the supported material suggests the coexistence of zeolite framework signals and EPS-derived functional groups. Hydrodynamic size and zeta potential of zeolite-supported EPS-stabilized AgNPs. (**D**) DLS profile showing a main population at ~249 nm. (**E**) Zeta potential distribution showing a dominant negatively charged population centered at ~−9.40 mV.

To investigate the interactions among the EPS matrix, biogenic AgNPs, and zeolitic matrix, comparative FTIR analysis was conducted (Figure 3C). This analysis was used to identify functional groups involved in EPS-mediated nanoparticle stabilization and to evaluate spectral modifications associated with AgNP immobilization on activated zeolite. The activated zeolite spectrum showed the characteristic aluminosilicate framework, mainly represented by the intense region between 1200 and 950 cm^−1^, assigned to asymmetric T–O–T stretching vibrations, where T corresponds to Si or Al. On the other hand, bands near 790 cm^−1^ and 460 cm^−1^ can be associated with Si–O–Si stretching and T–O framework bending, respectively, confirming the acid activation process of the zeolitic structure without any change in the principal structure, as also studied by Badvi and Javanbakht [39].

The spectrum of EPS-stabilized AgNPs displayed characteristic features consistent with a polysaccharide-rich stabilizing organic layer, as previously reported by Banerjee et al. [40]. Broad contributions around 3400 cm^−1^ were attributed to O–H/N–H stretching vibrations, while the band near 1629–1637 cm^−1^ is associated with the -C=C- stretch of alkenes or the -C=O- stretch of amides. This region is particularly relevant because changes in its position and intensity after nanoparticle formation suggest the participation of carbonyl, carboxylate, amide, or water-associated groups in Ag^+^ binding, reduction, and subsequent stabilization of AgNP surfaces. [41,42]. A broad stretching vibration, near 2920–2850 cm^−1^, was assigned to C–H stretching, indicating the presence of hydroxyl- and aliphatic-rich structures associated with the EPS matrix. Signals in the 1420–1450 cm^−1^ region may also be associated with C–H bending or symmetric carboxylate-related vibrations, supporting the possible contribution of negatively charged EPS groups to metal interaction. Furthermore, the carbohydrate fingerprint region between 1200 and 950 cm^−1^, associated with C–O–C and C–O vibrations from glycosidic and hydroxyl-containing structures, showed spectral modifications consistent with the involvement of polysaccharide domains in AgNP capping. These spectral changes suggest that EPSs do not act only as a passive coating but rather as a multifunctional stabilizing matrix. The observed band shifts and intensity variations after AgNP formation support the formation of an EPS-derived capping layer, which may provide both steric and electrostatic stabilization. After immobilization onto activated zeolite, the FTIR profile of the Zeo-AgNP material retained the main aluminosilicate framework bands, confirming that the zeolitic structure was not disrupted during nanoparticle loading. At the same time, the supported material displayed spectral contributions attributable to EPS-derived functional groups. The overlap between the zeolitic T–O–T vibrations and EPS-associated C–O–C/C–O vibrations in the 1200–950 cm^−1^ region suggests the coexistence of the inorganic zeolite framework and the EPS-stabilized AgNP phase within the hybrid material. In addition, changes in the relative intensity and position of bands associated with O–H/N–H, carbonyl/carboxylate/amide, and carbohydrate vibrations after immobilization indicate interactions between the EPS-capped AgNPs and the zeolitic surface. These interactions may involve hydrogen bonding, electrostatic interactions, and/or coordination between EPS functional groups and surface silanol/aluminol sites of the zeolite, as seen in a similar study conducted by Badvi and Javanbakht [39]. Overall, the FTIR results support the formation of zeolite-immobilized AgNPs, in which the zeolitic framework remains structurally preserved while EPS-derived functional groups likely contribute to nanoparticle stabilization and interaction with the support.

#### 3.3.3. Hydrodynamic Diameter, PDI and Zeta Potential Analysis

The DLS and zeta potential analyses provided complementary information on the colloidal behavior and surface charge of the Zeo-AgNPs. The hydrodynamic size distribution showed a dominant population centered at ~249 nm (Figure 3D), which was slightly larger than that observed for free EPS-stabilized AgNPs. This increase is expected because the DLS measurement not only reflects the AgNPs but also the zeolite support and the surrounding EPS coating, resulting in a larger apparent hydrodynamic diameter. The predominance of a single major DLS population suggests that the zeolite-supported material formed a relatively defined dispersed fraction under the measurement conditions, without extensive multimodal aggregation across the analyzed range. Moreover, a single dominant DLS value suggests a relatively dispersed distribution under controlled conditions within the analyzed nanoscale range. This interpretation was further supported by a relatively low PDI value of 0.170 ± 0.032, indicating a relatively narrow and reproducible hydrodynamic distribution, with a monodisperse nature. In terms of homogeneity, the PDI value suggest uniform dispersed condition rather than an uncontrolled aggregated condition. Moreover, the PDI value is also associated with improved colloidal stability [43]. This behavior is also consistent with the SEM micrographs, where Ag-containing particulate domains appeared in association with the zeolitic surface rather than forming completely uncontrolled aggregates. Therefore, the relatively narrow size distribution and estimated PDI value confirmed the immobilization to produce a hybrid particle, although the apparent hydrodynamic size confirms that the zeolite carrier strongly governs the DLS response.

The zeta potential distribution of Zeo-AgNPs showed a dominant negatively charged population, with a peak centered at approximately −9.40 mV (Figure 3E). This negative surface charge suggests the retention of AgNPs along with the electrokinetic contribution of the EPS coating after immobilization onto the zeolitic support. The presence of anionic functional groups in the EPSs, including carboxylate, hydroxyl, and uronic acid residues, may contribute to the observed negative potential and supports the role of the biopolymeric layer in nanoparticle stabilization [44]. Compared with free EPS-stabilized AgNPs, which exhibited a more negative zeta potential, the less negative value observed after loading onto zeolite indicates surface charge modulation caused by an interaction between the EPS-coated nanoparticles and the aluminosilicate framework. This shift may be associated with partial charge screening, electrostatic interactions with exchangeable cations, or adsorption of AgNP/EPS complexes onto the zeolite surface. Importantly, the maintenance of a unimodal electrokinetic distribution suggests that the loaded system is dominated by a relatively homogeneous surface-charge population, without clear evidence of multiple electrokinetically distinct fractions.

Overall, the DLS and zeta potential profiles support the successful association of EPS-stabilized AgNPs with the zeolite matrix. The hydrodynamic size distribution, together with the estimated PDI value of 0.170 ± 0.032, confirms the formation of a relatively defined zeolite-governed hybrid particulate assembly, while the moderately negative zeta potential indicates that EPS-derived surface functionality remains detectable after immobilization. This behavior is relevant because particle size distribution and surface charge can influence dispersion, aggregation tendency, interaction with microbial cells, and the release or accessibility of silver-based active sites. Thus, the supported system combines the structural support of the zeolitic carrier with the stabilizing electrokinetic effect of the EPS layer, reinforcing its suitability as a multifunctional material for aqueous treatment or bioremediation and antimicrobial applications.

### 3.4. Evaluation of Antibacterial Activity and Determination of IC_90_ and MBC_99_

**Figure 4:**
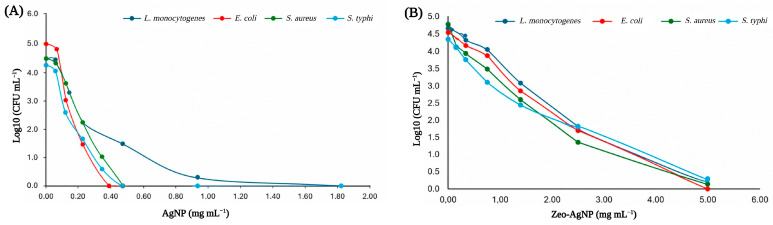
Concentration-dependent reduction in bacterial viability induced by (**A**) free EPS-stabilized AgNPs and (**B**) Zeo-AgNPs against representative Gram-positive and Gram-negative bacteria. Different concentration ranges were used for each system according to their experimentally determined antibacterial activity profiles.

The antibacterial behavior of free EPS-stabilized AgNPs and zeolite-supported AgNPs (Zeo-AgNPs) was evaluated against representative Gram-positive and Gram-negative bacteria through concentration–response assays, IC_90_, and MBC_99_ determinations (Figure 4A,B and Table 1). In both systems, bacterial viability progressively decreased as nanoparticle concentration increased, demonstrating a clear dose-dependent antimicrobial response [45]. However, free AgNPs produced a steeper reduction in viable cell counts than the zeolite-supported system, indicating greater antibacterial activity of dispersed nanoparticles in the colloidal suspension. In contrast, Zeo-AgNPs showed a more gradual reduction profile, suggesting that immobilization within the zeolitic matrix modulated the exposure and/or accessibility of Ag-associated active sites.

The quantitative data summarized in Table 1 confirmed this behavior. Free EPS-stabilized AgNPs exhibited IC_90_ values ranging from 0.055 to 0.123 mg mL^−1^ and MBC_99_ values between 0.098 and 0.283 mg mL^−1^, whereas Zeo-AgNPs required higher concentrations, with IC_90_ values ranging from 0.413 to 0.895 mg mL^−1^ and MBC_99_ values between 1.260 and 2.118 mg mL^−1^. These results indicate that zeolite immobilization attenuated the apparent short-term antibacterial potency of the nanoparticle system. This reduction should not be interpreted as a loss of intrinsic antimicrobial activity of the immobilized nanoparticles, but rather because of the supported configuration. In the free colloidal system, the nominal concentration more directly represents the amount of dispersed AgNPs available to interact with bacterial cells. Conversely, in Zeo-AgNPs, the applied concentration corresponds to the total hybrid material and not necessarily to the effective AgNP dose, since the actual nanoparticle loading and immobilization efficiency within the zeolitic matrix were not quantified. In addition, the mineral support may restrict nanoparticle mobility, reduce direct contact with bacterial cells, and modulate the local availability of Ag-associated antibacterial effect [46]. Therefore, comparisons between both systems should be interpreted as apparent antibacterial performance at the applied material-dose level.

Among the evaluated microorganisms, *S. typhi* was the most susceptible strain in both nanoparticle systems. Free AgNPs produced IC_90_ and MBC_99_ values of 0.055 and 0.098 mg mL^−1^, respectively, whereas Zeo-AgNPs reached 0.413 and 1.260 mg mL^−1^. *E. coli* also showed high susceptibility, with IC_90_/MBC_99_ values of 0.067/0.124 mg mL^−1^ for free AgNPs and 0.572/1.498 mg mL^−1^ for Zeo-AgNPs as similar kind of study done before also [47]. In contrast, *L. monocytogenes* exhibited the highest tolerance, particularly in the immobilized system, where the MBC_99_ value reached 2.014 mg mL^−1^. This differential response suggests a greater susceptibility of Gram-negative bacteria compared with Gram-positive strains, probably associated with differences in cell-envelope architecture and the accessibility of Ag-associated antibacterial effects. Overall, although zeolite immobilization reduced the apparent immediate antibacterial potency of the system, Zeo-AgNPs preserved relevant antibacterial activity across all tested strains, supporting their potential as a controlled and recoverable antibacterial material.

The differential antibacterial response suggests a greater susceptibility of Gram-negative bacteria compared with Gram-positive bacteria, probably due to differences in cell wall architecture. The thinner peptidoglycan layer and outer membrane organization of Gram-negative bacteria may facilitate the interaction of Ag-associated antimicrobial components, whereas the thicker and more compact peptidoglycan network of Gram-positive bacteria can partially restrict nanoparticle accessibility. Nevertheless, antibacterial activity of AgNPs is likely multifactorial, involving membrane disruption, oxidative stress generation, and Ag^+^ release.

The effect of immobilization was quantitatively evident when comparing free and supported systems. Relative to free AgNPs, Zeo-AgNPs required approximately 7.1-, 8.1-, 8.5-, and 7.5-fold higher IC_90_ values against *L. monocytogenes, S. aureus, E. coli*, and *S. typhi,* respectively. Similarly, MBC_99_ values increased by approximately 7.1-, 10.1-, 12.1-, and 12.8-fold, respectively. Despite this reduction in apparent short-term effect, antimicrobial activity was preserved after immobilization, indicating that the zeolitic matrix did not inactivate the EPS-stabilized AgNPs but rather modulated their immediate and controlled availability.

Importantly, according to the commonly used criteria that MBC/MIC ratios ≤ 4 indicate bactericidal activity [48], the MBC_99_/IC_90_ ratio remained below four for all strains in both systems. For free AgNPs, the ratio ranged from 1.782 to 2.301, whereas for Zeo-AgNPs it ranged from 2.295 to 3.053, supporting predominantly bactericidal rather than bacteriostatic response. Thus, although immobilization increased the concentrations required for bacterial inhibition, the supported system retained sufficient biological accessibility to induce irreversible bacterial damage.

From an applied perspective, free EPS-stabilized AgNPs showed greater immediate antibacterial efficiency, while Zeo-AgNPs may offer a more controlled and recoverable antimicrobial platform. This configuration could reduce uncontrolled nanoparticle dispersion, improve material retention, and moderate the availability of Ag-associated antimicrobial species. However, silver release behavior, reusability, long-term antimicrobial effect, and activity in real water matrices should be further evaluated to confirm the practical applicability of the zeolite-supported system.

### 3.5. Dye Removal Assay Using Zeo-AgNPs Against Methylene Blue (MB)

**Figure 5:**
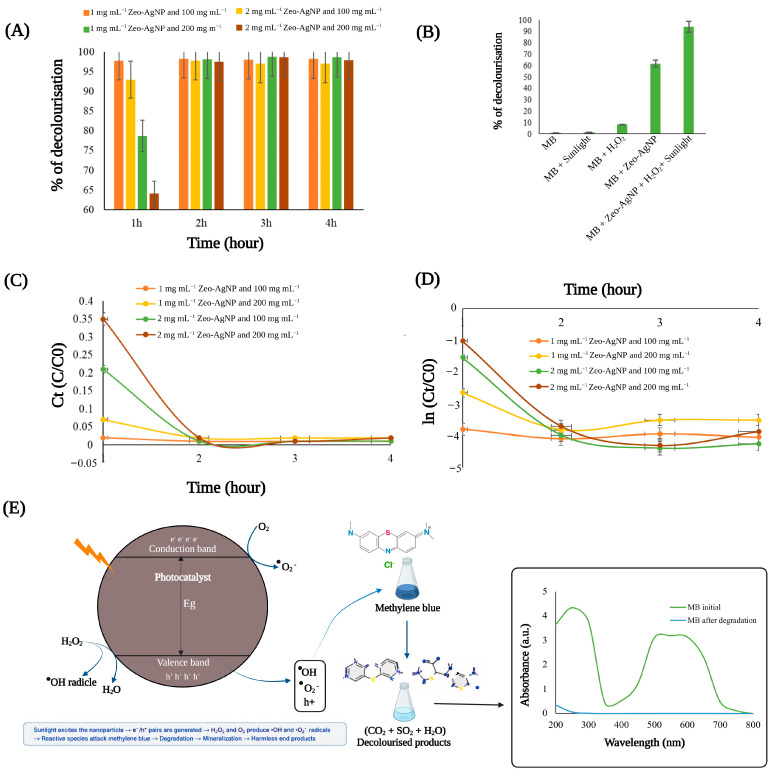
Photocatalytic degradation and kinetic analysis of methylene blue (MB) using Zeo-AgNPs under visible light irradiation and H_2_O_2_ treatment. (**A**) Percentage of MB degradation as a function of reaction time. (**B**) Decolorisation percentage of MB under control conditions, such as sunlight alone and H_2_O_2_ alone. (**C**) Kinetic degradation profile represented by Ct/C_0_ versus time. (**D**) Pseudo-first-order kinetic analysis represented by ln(Ct/C_0_) versus time. (**E**) Schematic illustration of the proposed photocatalytic mechanism of Zeo-AgNP-mediated MB decolorisation under visible light and H_2_O_2_ treatment, involving reactive oxygen species generation and dye degradation pathways. UV–Vis absorption spectra showing the reduction in MB absorbance intensity before and after treatment.

The dye removal from the aqueous solutions was carried out by hydrogen peroxide-assisted photocatalytic degradation methodology (Figure 5A). The present experiment was performed against 100 and 200 mg L^−1^ MB concentrations using two different concentrations of Zeo-AgNPs (1 mg ml^−1^ and 2 mg ml^−1^). The photocatalytic degradation of methylene blue using Zeo-AgNPs and H_2_O_2_ demonstrated a notable percentage of removal (Figure 5A). The present study showed maximum decolorization, 98.76 ± 0.08%, after 4 h of incubation under visible light of sunlight and H_2_O_2_ against 200 mg L^−1^ dye concentration. The same concentration of MB was evaluated under different reaction conditions to identify the most effective decolorization system. MB treated with sunlight alone and H_2_O_2_ alone exhibited only 1.49% and 8.33% decolorization, respectively, indicating that neither direct photolysis nor oxidation by H_2_O_2_ was sufficient for efficient dye removal. In contrast, treatment with Zeo-AgNPs alone (without H_2_O_2_ and sunlight) achieved 61.86% decolorization, which may be attributed to the combined adsorption capacity of zeolite and the catalytic activity of AgNPs. The highest decolorization efficiency (94.24%) was observed in the presence of Zeo-AgNPs, H_2_O_2_, and sunlight, demonstrating a synergistic photocatalytic effect through enhanced generation of reactive oxygen species responsible for rapid dye degradation (Figure 5B).

Furthermore, the reactions were considered for a kinetics study. For the best decolorization condition, the kinetic study (Figure 5C,D) indicates that the Zeo-AgNP-mediated MB decolorization follows pseudo-first-order kinetics with a rate constant (k) of 0.856 h^−1^. The degradation process follows a pseudo-first-order kinetic model, particularly in the initial phase, while deviations at later times suggest substrate (MB) depletion. It quantifies the efficiency of the degradation reaction, demonstrating a rapid conversion rate. The linear relationship $Ln\left(\frac{C_{t}}{C_{0}}\right)$ versus time confirms the applicability of the first-order kinetic model, consistent with nanoparticle-mediated photocatalysis of synthetic dyes. In comparison to other conventional physicochemical processes, green synthesized nanoparticle immobilized on natural zeolite provide a sustainable and environmentally friendly strategy for dye removal. At the time of decolorization, after 4 h, the absorbance at the peak (*λ*_*ma*x_) indicated the removal of the dye molecules. The change corresponds (Figure 5) to the reaction mixture’s visible transformations from vibrant blue to an almost colorless state. Therefore, both visual and spectroscopic analysis confirm the effective catalytic activity of the synthesized Zeo-AgNPs.

The mechanism of dye removal corresponds to the suppression of electron-hole recombination on the nano-photocatalyst surface by silver ions (Figure 5E). The nanocomposite promotes the decomposition of H_2_O_2_ into hydroxyl radicals through electron transfer reactions occurring on its surface. Under irradiation, electrons are excited from the valence band to the conduction band, generating electron-hole pairs. These reactive species interact with oxygen and water adsorbed on the surface, leading to the formation of hydroxyl radicals, as strong oxidizing agents (Figure 5E) [49]. In the H_2_O_2_-assisted photocatalytic process, light additionally breaks the peroxide bond of H_2_O_2_, producing more hydroxyl radicals and thereby enhancing the degradation efficiency [50]. Figure 5C,D show the concentration decay and kinetic investigations of the dye during the removal process at different times. During the degradation reaction, excited dye molecules may function as photosensitizers [51,52], transferring electrons to the conduction band [53] of the AgNPs. These transferred electrons subsequently react with dissolved molecular oxygen (O_2_), leading to the generation of reactive oxygen species such as superoxide anion radicals (O_2_^−^•) and hydroxyl radicals (OH•). Furthermore, H_2_O_2_ enhances the formation of free radicals, driving the oxidative degradation of dye molecules. There are several reports on plant material-based AgNPs as reducing agents and their catalytic activity against various organic pollutants. For example, biogenic AgNPs synthesized using *Moringa olifera* seeds showed good photocatalytic degradation against methylene blue (~81%) [54]. According to another study, AgNPs (~25.77 nm) synthesized from *Peltophorum pterocarpum* flower showed degradation (>90%) against acid blue 113 in the presence of NaBH_4_ within 20 min [31]. In contrast, microbial polysaccharide-stabilized AgNPs immobilized onto a natural zeolite matrix have been minimally investigated for their bioremediation capability. A limitation of the present study is that the total silver loading within the zeolite matrix and the corresponding Ag^+^ release kinetics were not directly quantified using advanced analytical techniques. Thus, future studies would focus on incorporating ICP-based analyses to determine silver loading efficiency, release behavior, and their contribution to the observed antimicrobial and physicochemical properties. Nevertheless, the present study showed the functionality of Tol1-EPS-stabilized AgNPs immobilized onto a zeolite matrix and their functionality towards the removal of synthetic dyes. Considering the above, this may be used in potential eco-friendly photocatalytic removal of synthetic dyes.

## 4. Conclusions

The present study successfully demonstrated the green synthesis of silver nanoparticles (AgNPs) using bacterial EPSs from *Bacillus licheniformis* Tol1 as a sustainable reducing, stabilizing, and capping agent. The subsequent immobilization of these biogenic AgNPs onto an acid-activated natural zeolite matrix resulted in a hybrid nanocomposite (Zeo-AgNP) that formed a well-defined hybrid particulate system while preserving the structural integrity of the zeolitic framework. Physicochemical characterization confirmed the successful incorporation of EPS-stabilized AgNPs within the zeolite support and indicated favorable structural and surface properties of the hybrid material. Functional assays revealed that the Zeo-AgNP system possesses potent, predominantly bactericidal activity against both Gram-positive (*S. aureus*, *L. monocytogenes*) and Gram-negative (*E. coli*, *S. typhi*) bacteria. Furthermore, it exhibited exceptional photocatalytic efficiency, achieving a ~98.65% degradation against methylene blue dye under solar irradiation within 4 h, following pseudo-first-order kinetics. Overall, the integration of a natural mineral support with EPS-mediated biogenic AgNPs generated a multifunctional material with promising antibacterial and photocatalytic properties. These findings highlight the potential of Zeo-AgNPs for environmental remediation applications. However, long-term stability, silver release, reusability, toxicity, and performance under realistic water treatment conditions were not evaluated in the present study and should be investigated in future research before practical implementation can be established.

## Figures and Tables

**Table 1:** Comparative antibacterial performance of free and zeolite-supported EPS-stabilized AgNPs.

Bacteria	mg mL^−1^				MBC_99_/IC_90_ Ratio	
	Free AgNPs		Zeo-AgNPs			
	IC_90_	MBC_99_	IC_90_	MBC_99_	Free AgNPs	Zeo-AgNPs
*L. monocytogenes*	0.123	0.283	0.878	2.014	2.301	2.295
*S. aureus*	0.111	0.209	0.895	2.118	1.883	2.365
*E. coli*	0.067	0.124	0.572	1.498	1.851	2.619
*S. Typhi*	0.055	0.098	0.413	1.260	1.782	3.053

IC_90_ and MBC_99_ values are expressed as mg mL^−1^. The MBC_99_/IC_90_ index was used to evaluate the predominance of bactericidal or bacteriostatic behavior of the nanoparticle system against each bacterial strain.

## Data Availability

The original contributions presented in this study are included in the article. Further inquiries can be directed to the corresponding authors.
